# Quality-adjusted survival in patients with metastatic colorectal cancer treated with fruquintinib plus best supportive care: results from FRESCO-2

**DOI:** 10.1016/j.esmoop.2025.104297

**Published:** 2025-02-21

**Authors:** S. Stintzing, J. Tabernero, T. Satoh, A. Dasari, S. Lonardi, C. Eng, R. Garcia-Carbonero, E. Elez, T. Yoshino, A.F. Sobrero, J.C. Yao, S. Kasper, D. Arnold, E. Basic, M. Granold, M. Petschulies, L. Wu, Y.-C. Chung, L. Chen, Z. Yang, E. Van Cutsem

**Affiliations:** 1 Charité – Universitätsmedizin Berlin, Corporate Member of Freie Universität Berlin and Humboldt Universität zu Berlin, Department of Hematology, Oncology and Cancer Immunology (CCM), Berlin, Germany; 2 Vall d’Hebron Barcelona Hospital Campus, Vall d’Hebron Institute of Oncology, Centro Cellex, Barcelona, Spain; 3 Department of Gastroenterological Surgery, Osaka University Graduate School of Medicine, Suita, Osaka, Japan; 4 Department of Gastrointestinal Medical Oncology, The University of Texas MD Anderson Cancer Center, Houston, USA; 5 Medical Oncology Unit 1, Veneto Institute of Oncology IOV-IRCCS, Padua, Italy; 6 Department of Medicine, Vanderbilt Ingram Cancer Center, Nashville, USA; 7 Oncology Department, Hospital Universitario 12 de Octubre, Madrid, Spain; 8 Department of Gastroenterology and Gastrointestinal Oncology, National Cancer Center Hospital East, Kashiwa, Japan; 9 Department of Medical Oncology, Azienda Ospedaliera San Martino, Genoa, Italy; 10 Department of Medical Oncology, West German Cancer Center, University Hospital Essen, Essen, Germany; 11 Department of Oncology and Hematology, Asklepios Tumorzentrum Hamburg, AK Altona, Hamburg, Germany; 12 Department of Business and Economics, Berlin School of Economics and Law, Berlin, Germany; 13 Takeda Pharma Vertrieb GmbH & Co. KG, Berlin, Germany; 14 Takeda Development Center Americas, Inc. (TDCA), Lexington, USA; 15 HUTCHMED International Inc., Florham Park, USA; 16 Gastroenterology/Digestive Oncology, University Hospitals Gasthuisberg/Leuven & KULeuven, Leuven, Belgium

**Keywords:** metastatic colorectal cancer, quality-adjusted survival, Q-TWiST, quality of life, VEGFR inhibitor

## Abstract

**Background:**

Treatment toxicity and disease-related symptoms of metastatic colorectal cancer (mCRC) can adversely affect quality of life (QoL). Maintaining QoL is an important treatment goal alongside improving survival outcomes. Quality-adjusted time without symptoms of disease or toxicity (Q-TWiST) measures the quality of patients’ survival by assessing the proportion of survival time that is free of symptoms/toxicity. The phase III FRESCO-2 study met its primary endpoint, demonstrating improved overall survival with fruquintinib plus best supportive care (BSC) versus placebo plus BSC [hazard ratio 0.66, 95% confidence interval (CI) 0.55-0.80, *P* < 0.001]. This *post hoc* Q-TWiST analysis compared the benefit–risk of fruquintinib versus placebo in all patients randomized in FRESCO-2.

**Methods:**

Patients with refractory mCRC in the USA, Europe, Japan, and Australia were randomized to receive fruquintinib (*n* = 461) or placebo (*n* = 230) plus BSC until disease progression or unacceptable toxicity. Patients’ survival time was partitioned as follows: time from randomization with grade 3/4 treatment-emergent adverse events (TEAEs) before progression (TOX); time from randomization to progression without grade 3/4 TEAEs (TWiST); and time from progression to death/censoring (REL). Q-TWiST was calculated as the combined utility-weighted mean durations of each health state, assuming utility coefficients of 1 for TWiST and 0.5 for TOX and REL.

**Results:**

Q-TWiST was improved when fruquintinib (versus placebo) was added to BSC, with a between-treatment difference of 2.0 months (95% CI 1.5-2.6 months, *P* < 0.05) and a relative improvement of 31.4%. This effect was primarily driven by the difference in the TWiST component [mean difference 2.1 months (95% CI 1.8-2.5 months), *P* < 0.05]. Q-TWiST improvements were consistent in all subgroups, including by age, sex, liver metastases, and primary tumor site. The subgroup and sensitivity analysis results confirmed the robustness of the primary analysis findings.

**Conclusions:**

Fruquintinib provides a clinically meaningful quality-adjusted survival benefit versus placebo in refractory mCRC.

## Highlights

• Q-TWiST measures the quality of patients’ survival by assessing the proportion of survival time without symptoms/toxicity.
• This *post hoc* Q-TWiST analysis of FRESCO-2 included all patients randomized to fruquintinib (*n* = 461) or placebo (*n* = 230).
• Q-TWiST was improved with fruquintinib vs placebo in the primary analysis (mean difference 2.0 months, 95% CI 1.5-2.6, *P* < 0.05).
• Primary analysis findings were robust, as demonstrated by the consistent results of subgroup and sensitivity analyses.
• Fruquintinib delays disease progression and prolongs patient survival without substantially increasing toxicity.

## Graphical abstract

**Figure:**
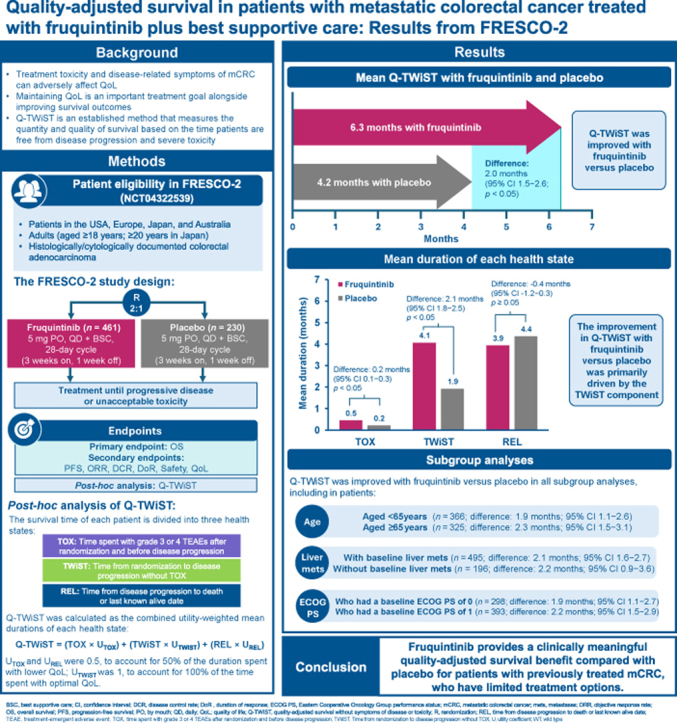

## Introduction

Metastatic colorectal cancer (mCRC) is associated with a poor prognosis, with current 5-year relative survival rates of ∼14%.^1^ Moreover, patients with mCRC suffer a high symptom burden.^2^, ^3^, ^4^ Severe disease-associated symptoms such as fatigue, constipation, diarrhea, and insomnia, in addition to related anxiety and depression, all affect patient quality of life (QoL),^2^^,^^3^ as do symptoms related to location of metastases and adverse events related to mCRC treatments.^4^, ^5^, ^6^ Consequently, there is a need for treatments that not only improve patient survival without compromising tolerability, but that also maintain or improve patient QoL.

Fruquintinib, a highly selective oral inhibitor of all three vascular endothelial growth factor receptors (VEGFRs -1, -2, and -3),^7^ was approved in China in September 2018 as a third or later line of therapy for mCRC,^8^ based on the results from the phase III FRESCO study (NCT02314819).^9^ FRESCO met its primary endpoint by demonstrating a significant overall survival (OS) benefit with fruquintinib versus placebo (median 9.3 versus 6.6 months, *P* < 0.001).^9^ To confirm the recommended phase II dose established in China, a bridging study was conducted in USA patients with advanced solid tumors.^10^^,^^11^ The global, phase III FRESCO-2 study (NCT04322539) investigated the efficacy and safety of fruquintinib combined with best supportive care (BSC) in a patient population that, unlike that in FRESCO, had received all standard cytotoxic and targeted therapies and had progressed on, or were intolerant to, trifluridine/tipiracil and/or regorafenib.^12^ FRESCO-2 met its primary endpoint by demonstrating improved OS with fruquintinib plus BSC compared with placebo plus BSC [median 7.4 versus 4.8 months, respectively, hazard ratio (HR) 0.66, 95% confidence interval (CI) 0.55-0.80, *P* < 0.001]. Additionally, fruquintinib was well tolerated in this heavily pretreated population, and demonstrated no deterioration of patient QoL.^12^^,^^13^ Based on these data and those from FRESCO, fruquintinib was approved in the USA in November 2023 for treatment of adult patients with mCRC who have been previously treated with fluoropyrimidine-, oxaliplatin-, and irinotecan-based chemotherapy, anti-VEGF therapy, and, if *RAS* wild type and medically appropriate, anti-epidermal growth factor receptor (EGFR) therapy.^14^ Fruquintinib was subsequently approved in June 2024 by the European Commission, based on FRESCO-2 data,^12^ for the treatment of adult patients with mCRC who have been previously treated with available standard therapies, including fluoropyrimidine-, oxaliplatin-, and irinotecan-based chemotherapies, anti-VEGF agents, anti-EGFR agents, and who have progressed on, or are intolerant to, treatment with either trifluridine/tipiracil or regorafenib.^15^

In the treatment of cancer, patients often need to make decisions regarding trade-offs between QoL and prognosis.^16^ Although cancer therapies can potentially prolong lengths of life, they may be associated with toxicities that impair QoL.^17^ There are numerous, well-validated, patient-reported questionnaires for measuring QoL, including in clinical trials.^18^ However, questionnaire responses are collected for a limited number of timepoints and tend to focus on specific disease features. As a result, while patient-reported QoL questionnaires provide a longitudinal view of a patient’s disease state at those timepoints, the sensitivity of such questionnaires to changes in overall patient QoL is limited, and dependent on the frequency of the timepoints and patient completion rates^19^; therefore, it can be difficult for patient-reported questionnaire data alone to inform discussions around treatment decisions. The quality-adjusted time without symptoms of disease or toxicity (Q-TWiST) is an established methodology that measures the quantity and quality of patients’ survival by assessing the proportion of survival time that is free of symptoms of progression or toxicity.^20^^,^^21^ It is a synthetic quality-adjusted life-year metric that is easy to use for the assessment of cancer treatments, as a supplement to patient-reported QoL. Q-TWiST can help to inform clinical decision making and regulatory agency approvals by integrating patient experiences with clinical data.^22^

This analysis used Q-TWiST methodology to compare the benefit–risk profile of fruquintinib plus BSC with that of placebo plus BSC among all patients who participated in the FRESCO-2 trial.

## Methods

### Study design and patients

Details of the FRESCO-2 study design and patient population have been reported previously.^12^ In summary, FRESCO-2 was a phase III, randomized, double-blind placebo-controlled study conducted in 14 countries globally (the USA, Europe, Asia, and Australia). Patients (aged ≥18 years; ≥20 years in Japan) enrolled in the study had documented mCRC, received all standard prior therapies including chemotherapy with fluoropyrimidine, oxaliplatin, and irinotecan, VEGF inhibitors, and EGFR inhibitors (if *RAS* wild type), and had disease progression on or were intolerant to trifluridine/tipiracil or regorafenib. Eligible patients were randomized according to a 2 : 1 ratio to receive fruquintinib 5 mg orally or matching placebo, given once daily on days 1-21 in 28-day cycles, plus BSC. Randomization was stratified by prior therapy with trifluridine/tipiracil, regorafenib, or both, *RAS* mutation status (wild type versus mutant), and duration of metastatic disease (≤18 months versus >18 months). Treatment continued until disease progression, death, unacceptable toxicity, patient withdrawal of consent, discontinuation by the physician, or study completion/termination. The primary endpoint of FRESCO-2 was OS, defined as the time from randomization to death from any cause. Secondary endpoints included progression-free survival (PFS), objective response rate, disease control rate, duration of response, safety, and patient-reported outcomes.

FRESCO-2 was conducted in accordance with the Declaration of Helsinki and Good Clinical Practice guidelines, including the International Council for Harmonisation of Technical Requirements for Pharmaceuticals for Human Use, and all applicable laws and regulations. The protocol was approved by the institutional review boards and independent ethics committees at each site.^12^ All participating patients provided written informed consent.

### Q-TWiST analysis

This was a *post hoc* Q-TWiST analysis based on individual-level patient data from all patients randomized in the FRESCO-2 study: fruquintinib plus BSC (*n* = 461) or placebo plus BSC (*n* = 230). The Q-TWiST calculation method has been described previously^23^^,^^24^; for the primary Q-TWiST analysis, the survival time for each patient was partitioned into three clinically relevant health states. Toxicity (TOX) is defined as the time spent with grade 3 or 4 treatment-emergent adverse events (TEAEs), including grade 3 or 4 laboratory abnormalities, after randomization and before disease progression (any day with multiple grade 3 or 4 TEAEs was only counted once). TWiST is the time from randomization to disease progression without toxicity. Relapse (REL) is defined as the time from disease progression to death or last known alive date. TOX, TWiST, and REL were calculated per patient by dividing the number of days in each health state by 30.437, to determine the duration of each in months. Each of the health states was assigned a utility coefficient, and the QoL-adjusted weighted sums of the mean duration of each health state was calculated to create the overall Q-TWiST score. TWiST was assigned the highest utility coefficient, as it was the time during which patients had optimal QoL. Lower utility coefficients were assigned for TOX and REL, as QoL was lower in these health states. The resulting quality-adjusted OS duration is shorter when QoL is not optimal.

As such, assuming a utility coefficient of 1 to account for 100% of the duration of TWiST (U_TWiST_) and 0.5 to account for 50% of the duration of TOX (U_TOX_) and REL (U_REL_), Q-TWiST was calculated as follows:

$$Q-\text{TWiST}=\left(\text{TOX}\;\times U_{\text{TOX}}\right)+\left(\text{TWiST}\;\times U_{\text{TWiST}}\right)+\left(\text{REL}\;\times U_{\text{REL}}\right)$$

The mean time spent in each of the health states was calculated for each treatment group using Kaplan–Meier estimation, and 95% CIs for the differences between treatment groups were calculated using the z-method (i.e. mean ± 1.96 × standard error), while nonparametric bootstrapping was used to calculate standard errors. Use of mean values was consistent with previous Q-TWiST analyses of the FRESCO and RECOURSE studies in patients with mCRC.^23^^,^^24^ The relative improvement (%) of Q-TWiST for the fruquintinib plus BSC group was calculated by dividing the Q-TWiST difference by the mean OS in the placebo plus BSC group. Relative Q-TWiST improvements of >10% implied a ‘clinically important’ difference while improvements of >15% suggested a ‘clearly clinically important’ difference.^21^ The analysis was performed with SAS software v9.4 (Cary, NC).^25^

### Subgroup and sensitivity analyses

Selected *post hoc* subgroup analyses were conducted, stratified by patient characteristics considered most relevant to disease progression and OS. These were: age (<65 years; ≥65 years; ≥70 years); sex (male; female); region (North America; Europe; Asia Pacific); Eastern Cooperative Oncology Group performance status (0; 1); primary tumor site at first diagnosis (colon, left; colon, right; colon, unknown; rectum only; colon, left and right); liver metastases at baseline (yes; no); number of metastatic sites (single; multiple); number of metastatic sites other than colon or rectum (single; multiple); duration of metastatic disease (≤18 months; >18 months); *RAS* status (wild type; mutant); *BRAF* status (wild type; V600E mutation; other); number of prior treatment lines for metastatic disease (≤3; >3); prior lines of chemotherapy (≤3; >3); prior VEGF inhibitors (yes; no); and prior trifluridine/tipiracil or regorafenib status (trifluridine/tipiracil; regorafenib; both trifluridine/tipiracil and regorafenib).

Considering that serious TEAEs can impact patients’ QoL and their ability to tolerate active treatments, a sensitivity analysis was conducted to ensure that the conclusion of the primary Q-TWiST analysis was robust in terms of toxicity. Q-TWiST was re-derived as the utility-weighted sum of the mean durations of TOX, TWiST, and REL, with TOX defined as the time spent with any serious TEAEs, instead of grade 3 or 4 TEAEs as used in the primary analysis.

## Results

### Baseline demographics and disease characteristics

Between August 2020 and December 2021, 691 patients were enrolled from 124 hospitals and cancer centers across 14 countries (North America, Europe, Asia, and Australia) and were randomized to receive fruquintinib plus BSC (*n* = 461) or placebo plus BSC (*n* = 230). Baseline demographics and disease characteristics were generally balanced between treatment arms and across the specific patient subgroups deemed most relevant to disease progression and patient survival (Table 1).

**Table 1:** Baseline demographics and disease characteristics

Characteristic	Fruquintinib plus BSC (*n* = 461)	Placebo plus BSC (*n* = 230)
**Age, years**		
<65	247 (53.6)	119 (51.7)
≥65	214 (46.4)	111 (48.3)
≥70	126 (27.3)	55 (23.9)
**Sex, *n* (%)**		
Male	245 (53.1)	140 (60.9)
Female	216 (46.9)	90 (39.1)
**ECOG PS score, *n* (%)**		
0	196 (42.5)	102 (44.3)
1	265 (57.5)	128 (55.7)
**Primary tumor site at first diagnosis, *n* (%)**		
Colon, left	192 (41.6)	92 (40.0)
Colon, right	97 (21.0)	53 (23.0)
Colon, unknown	25 (5.4)	13 (5.7)
Rectum only	143 (31.0)	70 (30.4)
Colon, left and right	4 (0.9)	2 (0.9)
**Liver metastases, *n* (%)**		
Yes	339 (73.5)	156 (67.8)
No	122 (26.5)	74 (32.2)
**Number of metastatic sites, *n* (%)**		
Single	61 (13.2)	41 (17.8)
Multiple	400 (86.8)	189 (82.2)
**Number of metastatic sites other than colon or rectum, *n* (%)**		
Single	61 (13.2)	44 (19.1)
Multiple	400 (86.8)	185 (80.4)
**Duration of metastatic disease, months, *n* (%)**		
≤18	37 (8.0)	13 (5.7)
>18	424 (92.0)	217 (94.3)
***RAS* status, *n* (%)**		
Wild type	170 (36.9)	85 (37.0)
Mutant	291 (63.1)	145 (63.0)
***BRAF* status, *n* (%)**		
Wild type	401 (87.0)	198 (86.1)
V600E mutation	7 (1.5)	10 (4.3)
Other	53 (11.5)	22 (9.6)
**No. prior chemotherapy treatment lines in metastatic disease, *n* (%)**		
≤3	125 (27.1)	64 (27.8)
>3	336 (72.9)	166 (72.2)
**Prior lines of chemotherapy, *n* (%)**		
≤3	77 (16.7)	44 (19.1)
>3	384 (83.3)	186 (80.9)
**Prior VEGF inhibitors, *n* (%)**		
Yes	445 (96.5)	221 (96.1)
No	16 (3.5)	9 (3.9)
**Prior trifluridine/tipiracil or regorafenib, *n* (%)**		
Trifluridine/tipiracil	240 (52.1)	121 (52.6)
Regorafenib	40 (8.7)	18 (7.8)
Trifluridine/tipiracil and regorafenib	181 (39.3)	91 (39.6)

BSC, best supportive care; ECOG PS, Eastern Cooperative Oncology Group performance status; VEGF, vascular endothelial growth factor.

### Q-TWiST analysis

**Figure 1:**
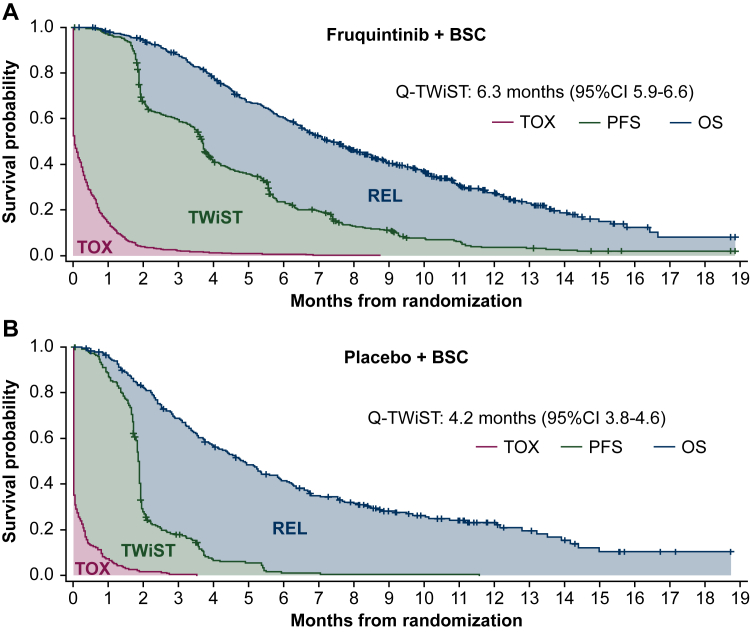
**Kaplan–Meier curves for OS, PFS, and toxicity in patients who received (A) fruquintinib plus BSC and (B) placebo plus BSC.** BSC, best supportive care; CI, confidence interval; OS, overall survival; PFS, progression-free survival; Q-TWiST, quality-adjusted time without symptoms of disease or toxicity; REL, relapse, defined as time from disease progression to death or censoring; TOX, toxicity, defined as the time spent with grade 3 or 4 treatment-emergent adverse events after randomization and before disease progression; TWiST, time without symptoms of disease or toxicity, defined as time from randomization to disease progression without toxicity.

**Figure 2:**
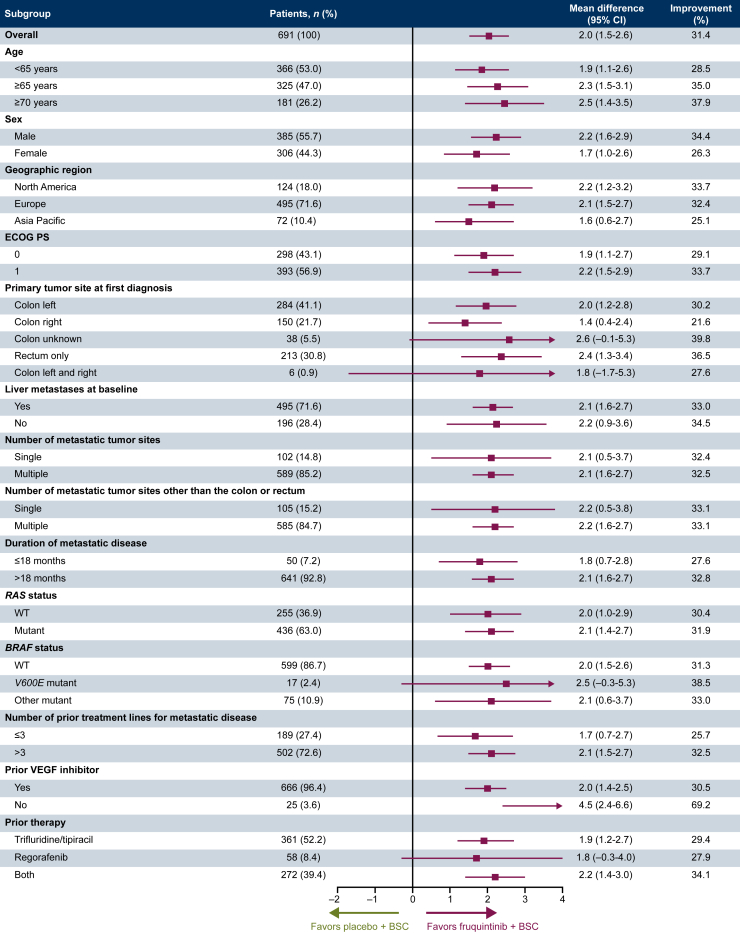
Differences in Q-TWiST by subgroup. BSC, best supportive care; CI, confidence interval; ECOG PS, Eastern Cooperative Oncology Group performance status; Q-TWiST, quality-adjusted time without symptoms of disease or toxicity; VEGF, vascular endothelial growth factor; WT, wild type.

The grade 3/4 TEAEs used to calculate Q-TWiST for each treatment group are included in Supplementary Table S1, available at https://doi.org/10.1016/j.esmoop.2025.104297. The Kaplan–Meier curves for OS, PFS, and toxicity for fruquintinib plus BSC and placebo plus BSC are shown in Figure 1A and B, respectively. The area between the curves illustrates the mean time that patients spent in each health state. The mean durations of Q-TWiST, TWiST, and TOX were longer when fruquintinib (versus placebo) was added to BSC, while the mean duration of REL was shorter (Table 2). The between-treatment difference in mean Q-TWiST was 2.0 months (95% CI 1.5-2.6 months, *P* < 0.05), with a relative improvement of 31.4% in favor of fruquintinib-based treatment. This effect was primarily driven by the difference between treatment arms in the TWiST component [mean duration: 4.1 months (fruquintinib plus BSC) versus 1.9 months (placebo plus BSC), difference: 2.1 months (95% CI 1.8-2.5 months), *P* < 0.05]. Consistent improvements in Q-TWiST were observed with fruquintinib plus BSC compared with placebo plus BSC across all subgroups included in the analysis (Figure 2).

**Table 2:** Mean duration of health states

	Mean duration, months (95% CI)	
Health state	Fruquintinib plus BSC (*n* = 461)	Placebo plus BSC (*n* = 230)
**Overall survival**	8.4 (8.0-8.9)	6.5 (5.8-7.2)
Difference (95% CI), *P* value	1.9 (1.1-2.8), <0.05	
**Progression-free survival**	4.5 (4.2-4.8)	2.1 (2.0-2.3)
Difference (95% CI), *P* value	2.4 (2.0-2.7), <0.05	
**Q-TWiST**	6.3 (5.9-6.6)	4.2 (3.8-4.6)
Difference (95% CI), *P* value	2.0 (1.5-2.6), <0.05	
**TWiST**	4.1 (3.8-4.4)	1.9 (1.8-2.1)
Difference (95% CI), *P* value	2.1 (1.8-2.5), <0.05	
**TOX**	0.5 (0.4-0.5)	0.2 (0.2-0.3)
Difference (95% CI), *P* value	0.2 (0.1-0.3), <0.05	
**REL**	3.9 (3.6-4.3)	4.4 (3.8-5.0)
Difference (95% CI), *P* value	−0.4 (−1.2 to 0.3), ≥0.05	

BSC, best supportive care; CI, confidence interval; Q-TWiST, quality-adjusted time without symptoms of disease or toxicity; REL, relapse, defined as time from disease progression to death or censoring; TWiST, time without symptoms of disease or toxicity, defined as time from randomization to disease progression without toxicity; TOX, toxicity, defined as the time spent with grade 3 or 4 treatment-emergent adverse events after randomization and before disease progression.

The results of the sensitivity analysis using serious TEAEs instead of grade 3/4 TEAEs to calculate TOX demonstrated the robustness of the primary analysis. The mean Q-TWiST was 6.4 months (95% CI 6.0-6.8 months) with fruquintinib plus BSC versus 4.3 months (95% CI 3.9-4.7 months) with placebo plus BSC. The between-treatment difference in mean Q-TWiST was 2.1 months (95% CI 1.6-2.7, months, *P* < 0.05) and there was a relative improvement of 33.0% in favor of the fruquintinib arm.

## Discussion

Q-TWiST analysis is used to measure the clinical benefit of an intervention by calculating the quality-adjusted time without symptoms of disease or toxicity, which is the time that is most important to patients with late-stage cancer.^26^ Q-TWiST analyses are becoming a common method to estimate the QoL impact of interventions on the survival time of patients with cancer, in addition to traditional QoL measurements. This *post hoc* analysis demonstrated a statistically significant and clinically meaningful Q-TWiST improvement of 2.0 months with fruquintinib plus BSC versus placebo plus BSC (6.3 versus 4.2 months, 95% CI 1.5-2.6 months, *P* < 0.05) in this heavily pretreated patient population with mCRC. The resulting relative Q-TWiST improvement of 31.4% was well above the 15% threshold commonly used to indicate a ‘clearly clinically important’ improvement.^21^ This effect was driven mainly by the difference in the TWiST component, or the time without symptoms of disease or toxicity. The Q-TWiST improvement with fruquintinib compared with placebo was consistent across all subgroups analyzed, including in patients aged <65, ≥65, and ≥70 years. The primary Q-TWiST analysis was also robust, as demonstrated by the sensitivity analysis.

In FRESCO-2, fruquintinib plus BSC was well tolerated with a safety profile that was consistent with the monotherapy profile established in FRESCO,^9^^,^^12^ and no new safety concerns were identified. Moreover, in this analysis, the time patients spent with grade 3 or 4 TEAEs (i.e. in the TOX health state) was relatively short with fruquintinib-based treatment. As such, this Q-TWiST analysis showed that fruquintinib delayed disease progression and prolonged patient survival without substantially increasing toxicity, which is particularly notable when considering that the toxicity was evaluated against an inactive placebo comparator. In the current analysis, for which we assumed a utility coefficient of 1 to account for 100% of the duration of TWiST (U_TWiST_) and 0.5 to account for 50% of the duration of TOX (U_TOX_) and REL (U_REL_), the difference in the duration of Q-TWiST compared with mean OS can be considered as an approximate indication of the impact that toxicities had on the quality of patients’ survival; a larger difference indicates that more time was spent in TOX and REL. Notably, in the fruquintinib group, the mean duration of Q-TWiST was 6.3 months, and mean OS was 8.4 months (difference: 1.9 months). In the placebo group, the mean duration of Q-TWiST was 4.2 months, and mean OS was 6.5 months (difference: 2.3 months). These data demonstrate that the quality of patients’ survival was less negatively impacted by grade 3/4 toxicities and symptoms of disease with fruquintinib compared with placebo.

Findings from this analysis, conducted in a multinational patient population, are consistent with a previous Q-TWiST analysis of the FRESCO study (NCT02314819) conducted in China. In FRESCO, patients with mCRC in China were randomized to receive daily oral fruquintinib or matching placebo, both plus BSC, until disease progression or unacceptable toxicity; the analysis reported a significant and clinically meaningful improvement in Q-TWiST of 7.01 versus 4.78 months [difference: 2.23 months (95% CI 1.41-3.04 months), *P* < 0.05] in the fruquintinib arm versus the placebo arm, with a clearly clinically important relative gain of 28.3%.^27^ Findings of the current analysis are also consistent with QoL assessments from FRESCO-2, which demonstrated that QoL was not negatively impacted with fruquintinib plus BSC versus placebo plus BSC according to patient-reported quality of life questionnaire (QLQ)-C30 global health status/QoL and EuroQol-5 dimension visual analog scale (EQ-5D-5L VAS) module results.^13^ The proportion of patients with stable or improved QoL after three cycles of treatment was similar or higher with fruquintinib plus BSC versus placebo plus BSC, while the median time to deterioration of QoL was longer with fruquintinib plus BSC versus placebo plus BSC.^13^ The results of this Q-TWiST analysis, alongside patient-reported outcomes from the FRESCO-2 study, are particularly notable considering that the patients included were heavily pretreated with a poor prognosis.^13^

Results of the primary Q-TWiST analysis appear favorable in the context of a Q-TWiST analysis of the RECOURSE study (NCT01607957), which investigated daily trifluridine/tipiracil versus placebo in a global cohort of patients with refractory mCRC; Q-TWiST was 5.48 months with trifluridine/tipiracil and 3.98 months with placebo (difference: 1.5 months), with a relative gain of ∼25%.^24^ It is important to note that the RECOURSE Q-TWiST analysis only investigated specific grade 3/4 TEAEs (nausea, vomiting, diarrhea, asthenia, anorexia, and febrile neutropenia),^24^ while this analysis of FRESCO-2 assessed all grade 3/4 TEAEs. The apparent difference in Q-TWiST between the current study and the RECOURSE trial may be a result of the difference in the safety profile with fruquintinib versus trifluridine/tipiracil. For example, treatment with trifluridine/tipiracil has been associated with a high incidence of myelosuppression,^28^^,^^29^ with 67% of patients who received trifluridine/tipiracil in the RECOURSE trial reporting neutropenia^30^; in contrast, <5% of patients who received fruquintinib experienced any grade all-cause or treatment-related neutropenia in FRESCO-2.^12^

Patient QoL outcomes with fruquintinib also seem generally favorable compared with regorafenib. For example, the CORRECT study (NCT01103323) investigated daily oral regorafenib versus placebo in a global cohort of patients with refractory mCRC. While CORRECT did not publish a Q-TWiST analysis, regorafenib has been associated with a high incidence of fatigue, palmar–plantar erythrodysesthesia, diarrhea, and hypertension^31^^,^^32^ which were experienced by 63.4%, 47.0%, 42.8%, and 30.4% of patients who received regorafenib in CORRECT, respectively.^33^ Nonetheless, there was no difference in patient-reported QoL with regorafenib versus placebo, as measured by the QLQ-C30 and EQ-5D-5L modules.^33^ In the phase II ReDOS regorafenib dose escalation study (NCT02368886), which was designed to investigate the standard regorafenib dose as well as an escalating dose to optimize efficacy and manage toxicity, the primary endpoint was met by demonstrating a significantly increased proportion of patients entering treatment cycle 3 in the dose escalation group versus the standard dose group (43% versus 26%, *P* = 0.043). However, QoL scores at the second week of treatment for current fatigue and activity interference (general, mood, walking ability, and work) were significantly worse for patients receiving the standard dose versus dose escalation regimen.^34^

### Limitations of this analysis

This Q-TWiST analysis was an unplanned, *post hoc* investigation of FRESCO-2. The Q-TWiST calculation used in this analysis was limited to investigating the time that patients experienced grade 3/4 and serious TEAEs, hence the impact of lower grade TEAEs on patient QoL was not assessed. Notably, standard utilities were used that did not necessarily reflect the underlying condition of the patients, as the methods used to determine the utility coefficients for the Q-TWiST calculation are not yet validated. It should be noted, however, that reference threshold analyses were conducted as a part of the Q-TWiST analysis of the FRESCO study, which indicated that patients who received fruquintinib plus BSC had a Q-TWiST benefit across all combinations of utility weights versus placebo plus BSC.^27^ A notable limitation with Q-TWiST analyses in general is that the cumulative QoL impact of TEAEs that occurred simultaneously cannot be taken into account using the methodology.

### Conclusions

The Q-TWiST analysis can evaluate trade-offs between potential treatment toxicities and survival time, which is clinically important for treatment decision making in later-line mCRC for patients whose QoL has been worsened by their disease and the prior therapies received. For such a patient population, it is important that a range of treatment options are available that provide a survival benefit without impacting QoL. In this analysis, fruquintinib plus BSC demonstrated a significant and clinically meaningful improvement in Q-TWiST versus placebo plus BSC. The Q-TWiST improvement was seen in all subgroups analyzed, including by age, and the primary analysis was robust, as demonstrated by the results of the sensitivity analysis. The totality of these data suggest that fruquintinib provides a clinically meaningful quality-adjusted survival benefit compared with placebo, for elderly as well as younger patients with previously treated mCRC who have limited treatment options.
