# Supplementary material for: Quality-adjusted survival in patients with metastatic colorectal cancer treated with fruquintinib plus best supportive care: results from FRESCO-2
Source: ESMO Open. 2025 Feb 21;10(3):104297. doi: 10.1016/j.esmoop.2025.104297 (PMC11904580; doi:10.1016/j.esmoop.2025.104297)
Supplement: Supplementary Table S1 [file mmc1.docx]

| **Supplementary Table 1. Grade 3/4 TEAEs reported in FRESCO-2 (Safety population)** | | | | | | | | | | |
| --- | --- | --- | --- | --- | --- | --- | --- | --- | --- | --- |
| **System Organ Class**  Preferred term | **Fruquintinib + BSC (n=456)** | | | | **Placebo + BSC (n=230)** | | | |  |  |
|  | **Grade 3** | | **Grade 4** | | **Grade 3** | | **Grade 4** | |  |  |
| **Patients with at least 1 TEAE, n (%)** | **220** | **(48.2)** | **18** | **(3.9)** | **65** | **(28.3)** | **6** | **(2.6)** |  |  |
| **General disorders and administration site conditions** | **57** | **(12.5)** | **2** | **(0.4)** | **9** | **(3.9)** | **1** | **(0.4)** |  |  |
| Aesthenia | 34 | (7.5) | 1 | (0.2) | 8 | (3.5) | 1 | (0.4) |  |  |
| Disease progression | 1 | (0.2) | 0 |  | 0 |  | 1 | (0.4) |  |  |
| Fatigue | 18 | (3.9) | 0 |  | 2 | (0.9) | 0 |  |  |  |
| General physical health deterioration | 6 | (1.3) | 3 | (0.7) | 3 | (1.3) | 0 |  |  |  |
| Condition aggravated | 3 | (0.7) | 0 |  | 0 |  | 1 | (0.4) |  |  |
| Non-cardiac chest pain | 3 | (0.7) | 0 |  | 0 |  | 0 |  |  |  |
| Mucosal inflammation | 2 | (0.4) | 0 |  | 0 |  | 0 |  |  |  |
| Pyrexia | 2 | (0.4) | 0 |  | 0 |  | 0 |  | |  |
| Chest pain | 1 | (0.2) | 0 |  | 0 |  | 0 |  | |  |
| Discomfort | 1 | (0.2) | 0 |  | 0 |  | 0 |  | |  |
| Malaise | 1 | (0.2) | 0 |  | 0 |  | 0 |  | |  |
| Oedema peripheral | 1 | (0.2) | 0 |  | 0 |  | 0 |  | |  |
| Ulcer | 1 | (0.2) | 0 |  | 0 |  | 0 |  | |  |
| Generalised oedema | 0 |  | 0 |  | 1 | (0.4) | 0 |  | |  |
| Pain | 0 |  | 0 |  | 1 | (0.4) | 0 |  | |  |
| **Gastrointestinal disorders** | **70** | **(15.4)** | **6** | **(1.3)** | **30** | **(13)** | **1** | **(0.4)** | |  |
| Diarrhoea | 16 | (3.5) | 0 |  | 0 |  | 0 |  | |  |
| Abdominal pain | 14 | (3.1) | 0 |  | 7 | (3) | 0 |  | |  |
| Stomatitis | 8 | (1.8) | 0 |  | 1 | (0.4) | 0 |  | |  |
| Vomiting | 7 | (1.5) | 0 |  | 4 | (1.7) | 0 |  | |  |
| Intestinal obstruction | 5 | (1.1) | 1 | (0.2) | 4 | (1.7) | 1 | (0.4) | |  |
| Small intestinal obstruction | 4 | (0.9) | 1 | (0.2) | 1 | (0.4) | 0 |  | |  |
| Ileus | 1 | (0.2) | 2 | (0.4) | 6 | (2.6) | 0 |  | |  |
| Intestinal perforation | 2 | (0.4) | 0 |  | 0 |  | 0 |  | |  |
| Nausea | 3 | (0.7) | 0 |  | 2 | (0.9) | 0 |  | |  |
| Proctalgia | 2 | (0.4) | 1 | (0.2) | 0 |  | 0 |  | |  |
| Rectal haemorrhage | 3 | (0.7) | 0 |  | 0 |  | 0 |  | |  |
| Constipation | 2 | (0.4) | 0 |  | 0 |  | 0 |  | |  |
| Gastrointestinal haemorrhage | 2 | (0.4) | 0 |  | 0 |  | 0 |  | |  |
| Small intestinal perforation | 1 | (0.2) | 1 | (0.2) | 0 |  | 0 |  | |  |
| Subileus | 1 | (0.2) | 0 |  | 2 | (0.9) | 0 |  | |  |
| Abdominal pain lower | 1 | (0.2) | 0 |  | 0 |  | 0 |  | |  |
| Abdominal pain upper | 1 | (0.2) | 0 |  | 0 |  | 0 |  | |  |
| Ascites | 1 | (0.2) | 0 |  | 3 | (1.3) | 0 |  | |  |
| Colitis | 1 | (0.2) | 0 |  | 0 |  | 0 |  | |  |
| Gastric haemorrhage | 1 | (0.2) | 0 |  | 0 |  | 0 |  | |  |
| Gastric perforation | 1 | (0.2) | 0 |  | 0 |  | 0 |  | |  |
| Gastrointestinal perforation | 0 |  | 1 | (0.2) | 0 |  | 0 |  | |  |
| Gastrointestinal toxicity | 1 | (0.2) | 0 |  | 0 |  | 0 |  | |  |
| Haematemesis | 1 | (0.2) | 0 |  | 0 |  | 0 |  | |  |
| Large intestinal obstruction | 1 | (0.2) | 0 |  | 1 | (0.4) | 0 |  | |  |
| Large intestine perforation | 1 | (0.2) | 0 |  | 0 |  | 0 |  | |  |
| Oesophageal obstruction | 1 | (0.2) | 0 |  | 0 |  | 0 |  | |  |
| Oesophagitis | 1 | (0.2) | 0 |  | 0 |  | 0 |  | |  |
| Pancreatitis | 1 | (0.2) | 0 |  | 0 |  | 0 |  | |  |
| Pancreatitis acute | 1 | (0.2) | 0 |  | 0 |  | 0 |  | |  |
| Proctitis | 1 | (0.2) | 0 |  | 0 |  | 0 |  | |  |
| Rectal perforation | 1 | (0.2) | 0 |  | 0 |  | 0 |  | |  |
| Abdominal distension | 0 |  | 0 |  | 2 | (0.9) | 0 |  | |  |
| Enterovesical fistula | 0 |  | 0 |  | 1 | (0.4) | 0 |  | |  |
| Rectal stenosis | 0 |  | 0 |  | 1 | (0.4) | 0 |  | |  |
| Upper gastrointestinal haemorrhage | 0 |  | 0 |  | 2 | (0.9) | 0 |  | |  |
| **Vascular disorders** | **65** | **(14.3)** | **2** | **(0.4)** | **4** | **(1.7)** | **0** |  | |  |
| Hypertension | 61 | (13.4) | 1 | (0.2) | 2 | (0.9) | 0 |  | |  |
| Hypertensive crisis | 2 | (0.4) | 1 | (0.2) | 0 |  | 0 |  | |  |
| Deep vein thrombosis | 1 | (0.2) | 0 |  | 0 |  | 0 |  | |  |
| Embolism | 1 | (0.2) | 0 |  | 0 |  | 0 |  | |  |
| Venous thrombosis | 1 | (0.2) | 0 |  | 0 |  | 0 |  | |  |
| Haemorrhage | 0 |  | 0 |  | 1 | (0.4) | 0 |  | |  |
| Hypotension | 0 |  | 0 |  | 1 | (0.4) | 0 |  | |  |
| **Investigations** | **40** | **(8.8)** | **5** | **(1.1)** | **19** | **(8.3)** | **0** |  | |  |
| Alanine aminotransferase increased | 14 | (3.1) | 0 |  | 1 | (0.4) | 0 |  | |  |
| Blood bilirubin increased | 10 | (2.2) | 1 | (0.2) | 6 | (2.6) | 0 |  | |  |
| Aspartate aminotransferase increased | 10 | (2.2) | 0 |  | 3 | (1.3) | 0 |  | |  |
| Lipase increased | 4 | (0.9) | 2 | (0.4) | 0 |  | 0 |  | |  |
| Amylase increased | 4 | (0.9) | 1 | (0.2) | 0 |  | 0 |  | |  |
| Gamma-glutamyltransferase increased | 5 | (1.1) | 0 |  | 2 | (0.9) | 0 |  | |  |
| Blood alkaline phosphatase increased | 3 | (0.7) | 0 |  | 3 | (1.3) | 0 |  | |  |
| Weight decreased | 3 | (0.7) | 0 |  | 1 | (0.4) | 0 |  | |  |
| Ejection fraction decreased | 2 | (0.4) | 0 |  | 1 | (0.4) | 0 |  | |  |
| Activated partial thromboplastin time prolonged | 1 | (0.2) | 0 |  | 1 | (0.4) | 0 |  | |  |
| Blood creatinine increased | 0 |  | 1 | (0.2) | 0 |  | 0 |  | |  |
| C-reactive protein increased | 0 |  | 1 | (0.2) | 2 | (0.9) | 0 |  | |  |
| Electrocardiogram QT prolonged | 1 | (0.2) | 0 |  | 1 | (0.4) | 0 |  | |  |
| International normalised ratio increased | 1 | (0.2) | 0 |  | 1 | (0.4) | 0 |  | |  |
| Liver function test increased | 1 | (0.2) | 0 |  | 0 |  | 0 |  | |  |
| Blood lactate dehydrogenase increased | 0 |  | 0 |  | 1 | (0.4) | 0 |  | |  |
| Blood triglycerides increased | 0 |  | 0 |  | 1 | (0.4) | 0 |  | |  |
| Liver function test abnormal | 0 |  | 0 |  | 1 | (0.4) | 0 |  | |  |
| SARS-CoV-2 test positive | 0 |  | 0 |  | 1 | (0.4) | 0 |  | |  |
| **Metabolism and nutrition disorders** | **29** | **(6.4)** | **3** | **(0.7)** | **11** | **(4.8)** | **1** | **(0.4)** | |  |
| Decreased appetite | 11 | (2.4) | 0 |  | 3 | (1.3) | 0 |  | |  |
| Hypokalaemia | 4 | (0.9) | 2 | (0.4) | 0 |  | 0 |  | |  |
| Hypoalbuminaemia | 4 | (0.9) | 0 |  | 0 |  | 0 |  | |  |
| Hyponatraemia | 4 | (0.9) | 0 |  | 1 | (0.4) | 0 |  | |  |
| Dehydration | 2 | (0.4) | 0 |  | 0 |  | 0 |  | |  |
| Hyperlipasaemia | 1 | (0.2) | 1 | (0.2) | 0 |  | 0 |  | |  |
| Hypermagnesaemia | 2 | (0.4) | 0 |  | 0 |  | 0 |  | |  |
| Diabetes mellitus inadequate control | 1 | (0.2) | 0 |  | 0 |  | 0 |  | |  |
| Hyperamylasaemia | 0 |  | 1 | (0.2) | 0 |  | 0 |  | |  |
| Hyperglycaemia | 1 | (0.2) | 0 |  | 1 | (0.4) | 0 |  | |  |
| Hyperkalaemia | 1 | (0.2) | 0 |  | 1 | (0.4) | 0 |  | |  |
| Hyperphosphataemia | 1 | (0.2) | 0 |  | 0 |  | 0 |  | |  |
| Hypertriglyceridaemia | 1 | (0.2) | 0 |  | 0 |  | 1 | (0.4) | |  |
| Hypocalcaemia | 1 | (0.2) | 0 |  | 0 |  | 0 |  | |  |
| Hypomagnesaemia | 1 | (0.2) | 0 |  | 0 |  | 0 |  | |  |
| Hypophosphataemia | 1 | (0.2) | 0 |  | 0 |  | 0 |  | |  |
| Cell death | 0 |  | 0 |  | 1 | (0.4) | 0 |  | |  |
| Diabetes mellitus | 0 |  | 0 |  | 1 | (0.4) | 0 |  | |  |
| Hyperuricaemia | 0 |  | 0 |  | 1 | (0.4) | 0 |  | |  |
| Hypoglycaemia | 0 |  | 0 |  | 1 | (0.4) | 0 |  | |  |
| Hypovolaemia | 0 |  | 0 |  | 1 | (0.4) | 0 |  | |  |
| Malnutrition | 0 |  | 0 |  | 1 | (0.4) | 0 |  | |  |
| **Skin and subcutaneous tissue disorders** | **31** | **(6.8)** | **0** |  | **1** | **(0.4)** | **0** |  | |  |
| Palmar-plantar erythrodysaesthesia syndrome | 29 | (6.4) | 0 |  | 0 |  | 0 |  | |  |
| Blister | 1 | (0.2) | 0 |  | 0 |  | 0 |  | |  |
| Skin necrosis | 1 | (0.2) | 0 |  | 0 |  | 0 |  | |  |
| Skin toxicity | 1 | (0.2) | 0 |  | 0 |  | 0 |  | |  |
| Rash | 0 |  | 0 |  | 1 | (0.4) | 0 |  | |  |
| **Infections and infestations** | **23** | **(5.0)** | **2** | **(0.4)** | **11** | **(4.8)** | **1** | **(0.4)** | |  |
| Pneumonia | 7 | (1.5) | 0 |  | 1 | (0.4) | 0 |  | |  |
| Sepsis | 4 | (0.9) | 1 | (0.2) | 0 |  | 0 |  | |  |
| Urinary tract infection | 3 | (0.7) | 1 | (0.2) | 4 | (1.7) | 0 |  | |  |
| Abscess limb | 1 | (0.2) | 0 |  | 0 |  | 0 |  | |  |
| Biliary tract infection | 1 | (0.2) | 0 |  | 0 |  | 0 |  | |  |
| Bronchitis | 1 | (0.2) | 0 |  | 0 |  | 0 |  | |  |
| Bronchopulmonary aspergillosis | 1 | (0.2) | 0 |  | 0 |  | 0 |  | |  |
| COVID-19 pneumonia | 1 | (0.2) | 0 |  | 0 |  | 0 |  | |  |
| Cellulitis | 1 | (0.2) | 0 |  | 0 |  | 0 |  | |  |
| Clostridium difficile colitis | 1 | (0.2) | 0 |  | 0 |  | 0 |  | |  |
| Clostridium difficile infection | 1 | (0.2) | 0 |  | 0 |  | 0 |  | |  |
| Device related infection | 1 | (0.2) | 0 |  | 0 |  | 0 |  | |  |
| Empyema | 1 | (0.2) | 0 |  | 1 | (0.4) | 0 |  | |  |
| Enterobacter sepsis | 1 | (0.2) | 0 |  | 0 |  | 0 |  | |  |
| Fournier's gangrene | 0 |  | 1 | (0.2) | 0 |  | 0 |  | |  |
| Infection | 1 | (0.2) | 0 |  | 0 |  | 0 |  | |  |
| Infection susceptibility increased | 1 | (0.2) | 0 |  | 0 |  | 0 |  | |  |
| Paronychia | 1 | (0.2) | 0 |  | 0 |  | 0 |  | |  |
| Perirectal abscess | 1 | (0.2) | 0 |  | 0 |  | 0 |  | |  |
| Pyelonephritis | 1 | (0.2) | 0 |  | 0 |  | 0 |  | |  |
| Septic shock | 0 |  | 0 |  | 1 | (0.4) | 0 |  | |  |
| COVID-19 | 0 |  | 0 |  | 1 | (0.4) | 1 | (0.4) | |  |
| Coronavirus infection | 0 |  | 0 |  | 1 | (0.4) | 0 |  | |  |
| Urosepsis | 0 |  | 0 |  | 1 | (0.4) | 0 |  | |  |
| Wound infection bacterial | 0 |  | 0 |  | 1 | (0.4) | 0 |  | |  |
| Wound sepsis | 0 |  | 0 |  | 1 | (0.4) | 0 |  | |  |
| **Respiratory, thoracic and mediastinal disorders** | **20** | **(4.4)** | **1** | **(0.2)** | **8** | **(3.5)** | **0** |  | |  |
| Dyspnoea | 8 | (1.8) | 2 | (0.4) | 3 | (1.3) | 0 |  | |  |
| Pulmonary embolism | 5 | (1.1) | 0 |  | 0 |  | 0 |  | |  |
| Pleural effusion | 2 | (0.4) | 0 |  | 2 | (0.9) | 0 |  | |  |
| Pneumonitis | 2 | (0.4) | 0 |  | 1 | (0.4) | 0 |  | |  |
| Bronchospasm | 1 | (0.2) | 0 |  | 0 |  | 0 |  | |  |
| Cough | 1 | (0.2) | 0 |  | 1 | (0.4) | 0 |  | |  |
| Dyspnoea exertional | 1 | (0.2) | 0 |  | 0 |  | 0 |  | |  |
| Hydrothorax | 1 | (0.2) | 0 |  | 0 |  | 0 |  | |  |
| Hypoxia | 1 | (0.2) | 0 |  | 0 |  | 0 |  | |  |
| Pulmonary artery occlusion | 1 | (0.2) | 0 |  | 0 |  | 0 |  | |  |
| Atelectasis | 0 |  | 0 |  | 1 | (0.4) | 0 |  | |  |
| Bronchopleural fistula | 0 |  | 0 |  | 1 | (0.4) | 0 |  | |  |
| **Renal and urinary disorders** | **23** | **(5.0)** | **0** |  | **6** | **(2.6)** | **0** |  | |  |
| Proteinuria | 8 | (1.8) | 0 |  | 2 | (0.9) | 0 |  | |  |
| Acute kidney injury | 6 | (1.3) | 0 |  | 1 | (0.4) | 0 |  | |  |
| Hydronephrosis | 3 | (0.7) | 0 |  | 0 |  | 0 |  | |  |
| Urinary tract obstruction | 3 | (0.7) | 0 |  | 0 |  | 0 |  | |  |
| Ureteric obstruction | 2 | (0.4) | 0 |  | 0 |  | 0 |  | |  |
| Chronic kidney disease | 1 | (0.2) | 0 |  | 0 |  | 0 |  | |  |
| Haematuria | 1 | (0.2) | 0 |  | 1 | (0.4) | 0 |  | |  |
| Renal failure | 1 | (0.2) | 0 |  | 1 | (0.4) | 0 |  | |  |
| Vesicocutaneous fistula | 1 | (0.2) | 0 |  | 0 |  | 0 |  | |  |
| Renal impairment | 0 |  | 0 |  | 1 | (0.4) | 0 |  | |  |
| **Nervous system disorders** | **15** | **(3.3)** | **2** | **(0.4)** | **2** | **(0.9)** | **0** |  | |  |
| Syncope | 3 | (0.7) | 0 |  | 0 |  | 0 |  | |  |
| Epilepsy | 2 | (0.4) | 0 |  | 0 |  | 0 |  | |  |
| Spinal cord compression | 2 | (0.4) | 0 |  | 1 | (0.4) | 0 |  | |  |
| Brain oedema | 1 | (0.2) | 0 |  | 0 |  | 0 |  | |  |
| Cerebral haemorrhage | 1 | (0.2) | 0 |  | 0 |  | 0 |  | |  |
| Cerebral infarction | 1 | (0.2) | 0 |  | 0 |  | 0 |  | |  |
| Cerebrovascular accident | 0 |  | 1 | (0.2) | 0 |  | 0 |  | |  |
| Depressed level of consciousness | 1 | (0.2) | 0 |  | 0 |  | 0 |  | |  |
| Headache | 1 | (0.2) | 0 |  | 0 |  | 0 |  | |  |
| Hepatic encephalopathy | 1 | (0.2) | 0 |  | 0 |  | 0 |  | |  |
| Intracranial pressure increased | 1 | (0.2) | 0 |  | 0 |  | 0 |  | |  |
| Posterior reversible encephalopathy syndrome | 0 |  | 1 | (0.2) | 0 |  | 0 |  | |  |
| Quadriparesis | 1 | (0.2) | 0 |  | 0 |  | 0 |  | |  |
| Transient ischaemic attack | 1 | (0.2) | 0 |  | 0 |  | 0 |  | |  |
| Ischaemic stroke | 0 |  | 0 |  | 1 | (0.4) | 0 |  | |  |
| **Hepatobiliary disorders** | **13** | **(2.9)** | **2** | **(0.4)** | **10** | **(4.3)** | **1** | **(0.4)** | |  |
| Hypertransaminasaemia | 5 | (1.1) | 0 |  | 1 | (0.4) | 0 |  | |  |
| Hyperbilirubinaemia | 3 | (0.7) | 1 | (0.2) | 1 | (0.4) | 1 | (0.4) | |  |
| Biliary obstruction | 1 | (0.2) | 1 | (0.2) | 1 | (0.4) | 0 |  | |  |
| Cholangitis | 3 | (0.7) | 0 |  | 1 | (0.4) | 0 |  | |  |
| Hepatic failure | 2 | (0.4) | 0 |  | 1 | (0.4) | 0 |  | |  |
| Cholecystitis | 2 | (0.4) | 0 |  | 0 |  | 0 |  | |  |
| Jaundice | 2 | (0.4) | 0 |  | 4 | (1.7) | 0 |  | |  |
| Bile duct stenosis | 1 | (0.2) | 0 |  | 0 |  | 0 |  | |  |
| Bile duct stone | 1 | (0.2) | 0 |  | 0 |  | 0 |  | |  |
| Cholestasis | 1 | (0.2) | 0 |  | 1 | (0.4) | 0 |  | |  |
| Hepatic cytolysis | 1 | (0.2) | 0 |  | 0 |  | 0 |  | |  |
| Portal vein thrombosis | 1 | (0.2) | 0 |  | 0 |  | 0 |  | |  |
| Hepatic function abnormal | 0 |  | 0 |  | 1 | (0.4) | 0 |  | |  |
| **Musculoskeletal and connective tissue disorders** | **16** | **(3.5)** | **0** |  | **4** | **(1.7)** | **0** |  | |  |
| Back pain | 6 | (1.3) | 0 |  | 3 | (1.3) | 0 |  | |  |
| Arthralgia | 4 | (0.9) | 0 |  | 0 |  | 0 |  | |  |
| Muscular weakness | 3 | (0.7) | 0 |  | 0 |  | 0 |  | |  |
| Fistula | 2 | (0.4) | 0 |  | 0 |  | 0 |  | |  |
| Pain in extremity | 2 | (0.4) | 0 |  | 0 |  | 0 |  | |  |
| Groin pain | 1 | (0.2) | 0 |  | 0 |  | 0 |  | |  |
| Musculoskeletal chest pain | 1 | (0.2) | 0 |  | 0 |  | 0 |  | |  |
| Spinal pain | 0 |  | 0 |  | 1 | (0.4) | 0 |  | |  |
| **Neoplasms benign, malignant and unspecified (incl cysts and polyps)** | **5** | **(1.1)** | **0** |  | **4** | **(1.7)** | **0** |  | |  |
| Cancer pain | 1 | (0.2) | 0 |  | 0 |  | 0 |  | |  |
| Metastases to central nervous system | 1 | (0.2) | 0 |  | 3 | (1.3) | 0 |  | |  |
| Metastases to meninges | 1 | (0.2) | 0 |  | 0 |  | 0 |  | |  |
| Metastasis | 1 | (0.2) | 0 |  | 0 |  | 0 |  | |  |
| Tumour pain | 1 | (0.2) | 0 |  | 0 |  | 0 |  | |  |
| Colorectal cancer metastatic | 0 |  | 0 |  | 1 | (0.4) | 0 |  | |  |
| **Blood and lymphatic system disorders** | **7** | **(1.5)** | **0** |  | **9** | **(3.9)** | **2** | **(0.9)** | |  |
| Anaemia | 5 | (1.1) | 0 |  | 7 | (3.0) | 0 |  | |  |
| Polycythaemia | 1 | (0.2) | 0 |  | 0 |  | 0 |  | |  |
| Thrombocytopenia | 1 | (0.2) | 0 |  | 0 |  | 1 | (0.4) | |  |
| Leukocytosis | 0 |  | 0 |  | 3 | (1.3) | 0 |  | |  |
| Neutropenia | 0 |  | 0 |  | 0 |  | 1 | (0.4) | |  |
| **Injury, poisoning and procedural complications** | **6** | **(1.3)** | **0** |  | **0** |  | **0** |  | |  |
| Femur fracture | 3 | (0.7) | 0 |  | 0 |  | 0 |  | |  |
| Fall | 1 | (0.2) | 0 |  | 0 |  | 0 |  | |  |
| Joint injury | 1 | (0.2) | 0 |  | 0 |  | 0 |  | |  |
| Sternal fracture | 1 | (0.2) | 0 |  | 0 |  | 0 |  | |  |
| **Cardiac disorders** | **5** | **(1.1)** | **0** |  | **2** | **(0.9)** | **0** |  | |  |
| Atrial fibrillation | 2 | (0.4) | 0 |  | 0 |  | 0 |  | |  |
| Cardiac failure congestive | 2 | (0.4) | 0 |  | 1 | (0.4) | 0 |  | |  |
| Cardiac failure | 1 | (0.2) | 0 |  | 0 |  | 0 |  | |  |
| Tachycardia | 1 | (0.2) | 0 |  | 0 |  | 0 |  | |  |
| Acute myocardial infarction | 0 |  | 0 |  | 1 | (0.4) | 0 |  | |  |
| **Psychiatric disorders** | **5** | **(1.1)** | **0** |  | **1** | **(0.4)** | **0** |  | |  |
| Confusional state | 3 | (0.7) | 0 |  | 0 |  | 0 |  | |  |
| Anxiety | 1 | (0.2) | 0 |  | 0 |  | 0 |  | |  |
| Disorientation | 1 | (0.2) | 0 |  | 0 |  | 0 |  | |  |
| Depressed mood | 0 |  | 0 |  | 1 | (0.4) | 0 |  | |  |
| **Endocrine disorders** | 2 | (0.4) | 0 |  | 0 |  | 0 |  | |  |
| Hypothyroidism | 2 | (0.4) |  |  | 0 |  | 0 |  | |  |
| **Reproductive system and breast disorders** | **2** | **(0.4)** | **0** |  | **0** |  | **0** |  | |  |
| Female genital tract fistula | 1 | (0.2) | 0 |  | 0 |  | 0 |  | |  |
| Intermenstrual bleeding | 1 | (0.2) | 0 |  | 0 |  | 0 |  | |  |
| **Immune system disorders** | **1** | **(0.2)** | **0** |  | **0** |  | **0** |  | |  |
| Food allergy | 1 | (0.2) | 0 |  | 0 |  | 0 |  | |  |

Datacut: June 24^th^, 2022. AEs are coded using MedDRA version 25.0. Percentages are based on the number of subjects in the safety population of each treatment group. During the period from the date of first study drug administration until 37 days after the last study drug administration or initiation of a new treatment of anti-tumor therapy, whichever is earlier, an AE is considered a TEAE if the onset date is on or after the start of study treatment or if the onset date is missing, or if the AE has an onset date before the start of the study treatment but worsened in severity. After this period, treatment-related SAEs will also be considered as TEAEs. Subjects with more than one TEAE are counted once at the worst severity category. A subject with multiple TEAE entries in the same SOC (PT) is only counted once within a particular SOC (PT). Number (%) of subjects with TEAE, sorted by SOC followed by PT in decreasing order of frequency (by Fruquintinib column). If the frequencies tie, an alphabetic order will be applied.

AE, adverse event; BSC, best supportive care; PT, Preferred term; SOC, System organ class; TEAE, treatment-emergent AE
